# Broad neutralization against SARS-CoV-2 variants induced by a next-generation protein vaccine V-01

**DOI:** 10.1038/s41421-021-00350-6

**Published:** 2021-11-30

**Authors:** Shiyu Sun, Xi Chen, Jingjing Lin, Junwen Ai, Jiaming Yang, Zhenxiang Hu, Yang-Xin Fu, Hua Peng

**Affiliations:** 1 Institute of Biophysics, Chinese Academy of Sciences, Beijing, China; 2 LivzonBio Inc., Zhuhai, Guangdong China; 3 Department of Pathology, University of Texas Southwestern Medical Center, Dallas, TX USA; 4 Guangzhou Laboratory, and Bioland Laboratory (Guangzhou Regenerative Medicine and Health Guangdong Laboratory), Guangzhou, Guangdong China

**Keywords:** Innate immunity, Mechanisms of disease

Dear Editor,

The emergence and rapid transmission of severe acute respiratory syndrome coronavirus 2 (SARS-CoV-2) variants have led to serious concerns and social panic over increased transmissibility, disease severity, and the potential immune escape induced by the previous vaccination^1^. High titers of neutralizing antibodies after vaccination play a critical role in limiting viral spreading and alleviating disease severity. However, it has been challenging to generate effective vaccines for maintaining long-lasting high-titer neutralizing antibodies. The traditional alum-adjuvanted protein vaccines have short-term minor toxicity but do not generally induce robust T and B cell responses, while the increased efficacy with newer adjuvants is often correlated with increased toxicity and is thus unsuitable for preventive use. We recently developed a next-generation SARS-CoV-2 protein vaccine, an interferon (IFN)-armed receptor-binding domain (RBD)-dimer fusion protein vaccine (I-P-R-F or briefly named V-01). V-01 generates a high titer of neutralizing antibodies with low toxicity, resulting in complete anti-viral protection and even virus clearance at the upper respiratory tract 24 h after infection in vaccinated monkeys^2^. In the clinical trials, participants receiving V-01 presented 3- to 4-fold higher serum neutralizing antibody (nAb) titers to the original SARS-CoV-2 strain than convalescent sera. V-01 also demonstrated an excellent safety profile in both younger and even elder groups in phase I and phase II trials^3,4^, respectively.

Currently, significant efforts and resources have been invested to generate new vaccines, following the ongoing variants of concern (VOCs). However, VOCs are constantly evolving with unpredictable mutations. It was unclear whether the vaccine of the original strain could effectively protect the host against major VOCs, especially the Delta variant. Given that two low-dose V-01 (10 μg) induced significantly high titer nAbs in phase I/II clinical trials, we investigated the protective effect of V-01 against VOCs in the trials. Pseudovirus neutralization assays were performed with serum samples from the V-01 phase II trial participants collected on day 49 (28 days post the second dose) after two-dose vaccination. Impressively, the viral neutralization titers against B.1.1.7 (Alpha) and B.1.617.2 (delta) variants decreased less than two-fold (a ratio of wild-type (WT)/variant) in pseudovirus assay compared to WT SARS-CoV-2, while a 5.6-fold reduction was observed for B.1.351 (Beta), similar to that of the mRNA-vaccinated human sera^5^ (Fig. 1a). We also tested the neutralizing activity against Alpha- and Beta-variant pseudoviruses for sera from mice immunized with the two-dose mouse V-01 vaccine. Similar to the V-01 clinical data, the average neutralization titers against Alpha and Beta variants were reduced by only 1.3- and 2.0-fold, respectively, compared to WT virus (Supplementary Fig. S1a, b). These results suggest that high-titer nAb induced by V-01 vaccination may protect the vaccine recipients against both the original strain and the VOCs.

**Fig. 1:**
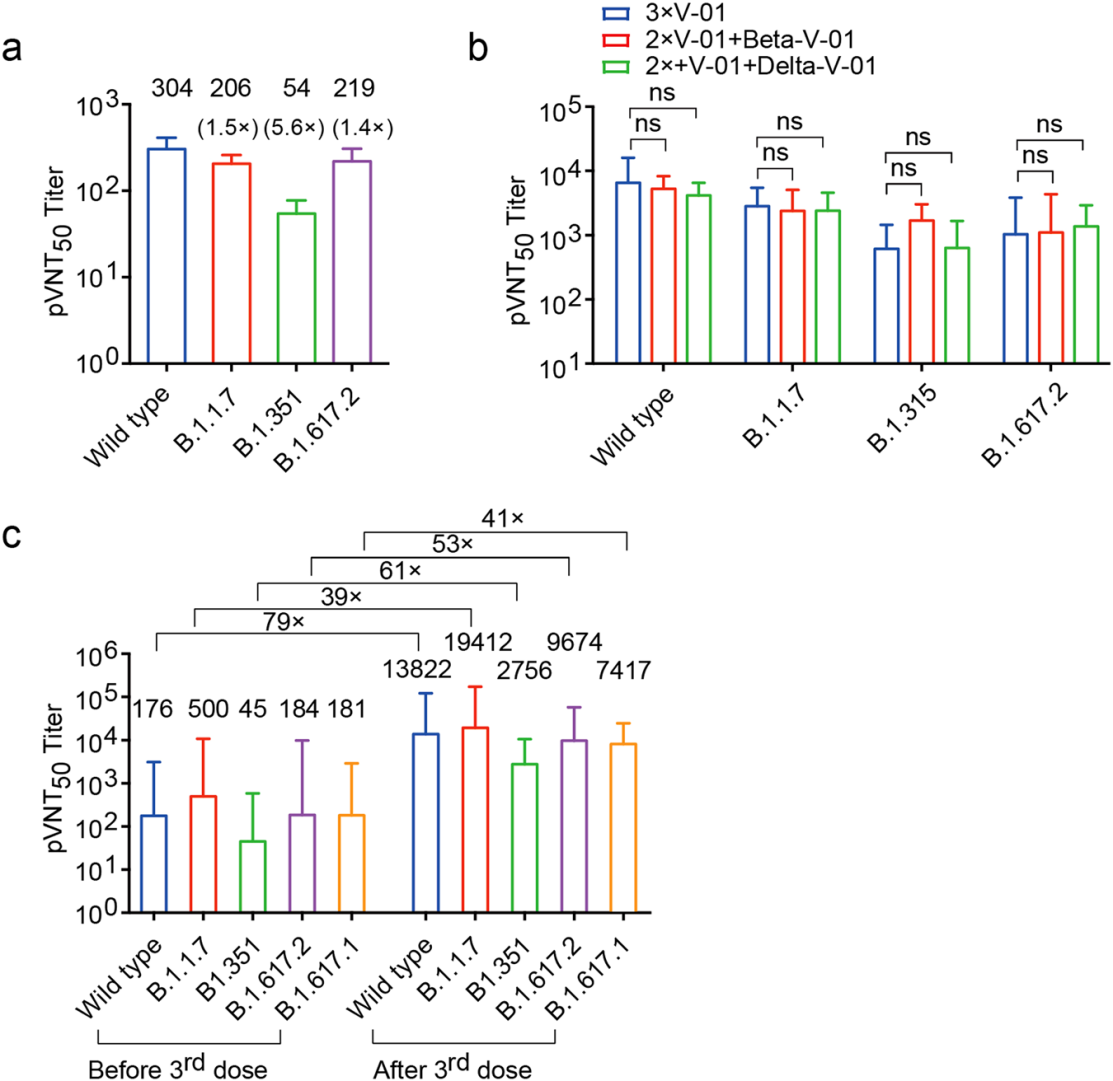
Analysis for neutralization of variant SARS-CoV-2 by serum samples from the V-01 phase II trial participants and the V-01-vaccinated animals. **a** Serum samples (*n* = 20) were collected from participants in the V-01 vaccine phase II trial on day 49 (28 days post the second dose) immunized with two doses of 10 μg of V-01. Samples were tested for neutralizing activity against WT SARS-CoV-2, B.1.1.7, B.1.351, and B.1.617.2 variants, using pseudovirus neutralization assays. The geometric mean of neutralizing antibody titer (GMT) is shown at the top of the indicated column, and 95% confidence intervals are shown. The GMT fold-reduction over the WT for each variant is indicated. **b** C57BL/6 mice (*n* = 10/group) were intramuscularly vaccinated three times with 2 μg of V-01 on days 0, 14, 28, or immunized twice with 2 μg of V-01 and further boosted with the equivalent dose of Beta-V-01 or Delta-V-01 on day 28 after prime immunization, respectively. The serum samples were collected on day 42 after initial vaccination to evaluate the neutralizing antibody titers. The pVNT_50_ in serum sample against wild-type SARS-CoV-2, B.1.315, B.1.617.2, and B.1.1.7 variants were analyzed using pseudovirus neutralization assays. **c** Rhesus macaques were intramuscularly immunized with V-01 via a prime-boost vaccination regimen at a 14-day interval. These macaques were then given a booster dose of V-01 after 300 days of initial vaccination. The serum samples were collected on day 300 post the initial immunization and 21 days after the third dose and subjected to pseudovirus neutralization assays. The pVNT_50_ of these sera against WT SARS-CoV-2 and the variants B.1.1.7, B.1.351, B.1.67.2, and B.1.67.1 was analyzed. The GMT fold changes after the third dose vs before the third dose are labeled. Data are shown as GMT and 95% confidence intervals. *P* values were calculated by one-way ANOVA with multiple comparison tests. n.s., not significant, *P < 0.05, **P < 0.01.

Recently, the COVID-19 vaccination booster administered after the initial two-dose vaccination has been advocated to achieve higher and prolonged nAb titers due to gradual reduction of protection in only a few months after the initial two doses^6,7^. Whether variant-modified vaccines should replace prototype vaccines for boosting immunization against the respective variants has been debated. To investigate vaccine potency to VOCs, we first tested the immunogenicity of a two-dose prototype of V-01, modified B.1.1.7 (Alpha-V-01), and B.1.351 (Beta-V-01) vaccines in mice. All vaccines can induce potent RBD-specific antibodies that bind to both the original RBD and RBD variants with no significant difference (Supplementary Fig. S1c). Delta-V-01 enhances the nAb titer to the Delta variant virus, just as the Alpha-V-01 enhances the nAb titer to the Alpha variant (Supplementary Fig. S1d, e). However, the serum nAb titers from Alpha-V-01- and Beta-V-01-vaccinated mice against the original SARS-CoV-2 strain drop significantly (Supplementary Fig. S1f, g), suggesting potentially narrow and selective protection by variant vaccines.

To investigate whether the booster of variant-modified vaccines (Beta or Delta) after the two-dose prototype V-01 immunization could induce more potent nAb to the respective variants than the prototype of V-01, we compared the nAb titer to the original strain, Beta, and Delta variants after booster vaccination in mice. Unexpectedly, the sera from all booster-vaccinated mice effectively cross-neutralized both the original SARS-CoV-2 strain and VOCs, including the Alpha, Beta, and Delta (Fig. 1b). This result indicates that booster immunization with the prototype of V-01 may be potent enough to neutralize the original as well as the Alpha, Beta, and Delta variants. A prototype vaccine booster is the timeliest and the most practical way to avoid the selection of new variants, as each new vaccine requires enormous time and effort to prepare, test, and approve. To mimic the clinical setting, we evaluated monkeys that received prime-boost vaccination (14 days apart) of V-01 300 days prior and showed reduced titers of nAbs over time (Supplementary Fig. S2). The macaques were then given a third dose of a prototype of V-01. Impressively, the third dose V-01 resulted in dramatically increased antibody levels in the macaques. The high titer neutralizing antibodies are protective against WT SARS-CoV-2 and all common VOC pseudoviruses available, including a 79-fold increase in nAbs for the prototype; 39-fold increase for the Alpha, B.1.1.7; 61-fold increase for Beta, B.1.351; 53-fold for Delta, B.1.617.2; 41-fold for Kappa, B.1.617.1 strains (Fig. 1c and Supplementary Fig. S2). These data indicate that polyclonal antibodies induced by the third dose of V-01 are sufficient to generate higher levels of neutralizing antibodies than those induced by the second dose and potently neutralize both the original virus as well as a broader range of VOCs.

Decreased vaccine protection against highly transmitted VOCs has been reported even in fully vaccinated hosts, especially a few months after vaccination^8^. Reduced immunity of vaccinated hosts could contribute to current viral transmission rates, notably the Delta variant^9^. High titers of neutralizing antibodies play a significant role in the control of disease severity and inhibition of deadly VOC generation due to extensive virus proliferation in millions of hosts. Developing COVID-19 variant vaccines could enhance nAb responses to the corresponding VOCs^10^. Nevertheless, rapidly evolving VOCs may overrun the manufacturing speed of safe VOC vaccines. Impressively, additional V-01 can boost the high titer of nAb to broad VOCs, comparable to the anti-variant analyses from the Moderna and Pfizer‐BioNTech vaccines^5,11^. We expect that V-01 could sustain prolonged protection, as we have observed that monkeys with a booster of prototype V-01 vaccine 300 days post the initial two doses generated much higher titer neutralizing antibodies against all known VOCs. These data fully suggest that robust memory immunity could be effectively preserved against all variants by a two-dose V-01 vaccination for an extended time and could be rapidly re-activated after the third dose with the original V-01, comparable to the variant-modified V-01, challenging the tremendous effort and resources to generate endless variant-based new vaccines. Therefore, V-01, the next-generation protein vaccine, could become a potent vaccine candidate to counter the future COVID-19 variants, especially for boosting the fading immunity after initial two-dose immunization.

## Supplementary information

Supplementary Information
