# Supplementary material for: Broad neutralization against SARS-CoV-2 variants induced by a next-generation protein vaccine V-01
Source: Cell Discov. 2021 Nov 30;7:114. doi: 10.1038/s41421-021-00350-6 (PMC8630016; doi:10.1038/s41421-021-00350-6)
Supplement: Supplementary file 1 — Supplementary Information [file 41421_2021_350_MOESM1_ESM.pdf]

Supplementary information

Methods and Materials

Animal immunization

The antigen was diluted with PBS for mouse immunization, and mixed with a fixed-dose (100 μg/dose) of alum adjuvant (SEVA, Germany). C57BL/6 mice obtained from Vital River (Beijing) were intramuscularly immunized with the antigen. Serum samples were collected at the indicated time points and used to perform the antibody assays.

Rhesus macaques were purchased from Guanxi Fangchenggang Biotechnology Development CO., Ltd. For rhesus macaque's immunization, a total of 3 adult macaques (weighing 3-5 kg) were immunized intramuscularly with V-01 and boosted with the same dose of V-01 on 14 and 300 days after the initial vaccination. Serum samples were collected at indicated time points. All the details of animals' vaccination were described in the figure legends.

ELISA

The 96-well ELISA plates (Conning, USA) were coated with 100 μL of wild-type RBD or variant RBD (1.5 μg/mL) overnight at 4 °C. Plates were washed with PBS and blocked with blocking buffer (PBS containing 5% fetal bovine serum, FBS). Serum samples were serially diluted and added to the blocked plates, followed by incubation at 37°C for 1 hour. Plates were then washed with PBST (PBS containing 0.05% Tween 20) and incubated with goat anti-mouse IgG-HRP (1:5000, Cwbiotech) at 37°C for 30 minutes. Plates were washed with PBST, and HRP substrate TMB was added. The reactions were stopped by 2M sulfuric acid. The absorbance at 450-630 was read using a microplate reader (Molecular Devices). The endpoint titers were defined as the reciprocal of maximum serum dilution at which the absorbance was higher than 2.5-fold of the background.

Pseudovirus neutralization assay

The SARS-CoV-2 wild type and variant pseudoviruses, including B.1.1.7, B.1.315, P1, B.1.617.1, B.1.617.2, and B.1.429, were purchased from Beijing Tiantan Pharmaceutical Biotechnology Development Co. Ltd. To evaluate the neutralization of vaccinated animal serum, 293-hACE2 cells were seeded into 96-well plates ($2x10^4$ per well) and incubated at 37 °C for 24 hours. Heat-inactivated serum samples were serially

diluted at 1:3 and incubated with 1000 $TCID_{50}$ of pseudovirus for 1 hour at 37°C. Medium mixed with pseudovirus was given as a control. The mixture was transferred to cells in the 96-well plates, and the plates were continued to incubate for another 24 hours. According to the manufacture's instruction, the luciferase substrate was added, and luciferase activity was determined by the Bright-LiteTM Luciferase Assay System (Vazyme). The 50% neutralization titer ($pVNT_{50}$) was defined as the reciprocal of serum dilution at which the relative light units (RUL) were reduced by 50% compared with the virus control wells.

Statistical analyses

All statistical analyses were performed using Graphpad Prism 8.0. Data are shown as the geometric mean with 95% confidence intervals. Statistical analyses were compared with an unpaired or paired student's two-tailed t-test for comparison between two groups. One-way ANOVA with Turkey's multiple comparisons test was used for comparisons among multiple groups. P values of < 0.05 were considered significant. $p<0.05$ (*), $p<0.01$ (**), $p<0.001$ (***) and $p<0.0001$ (****). ns, no significance.

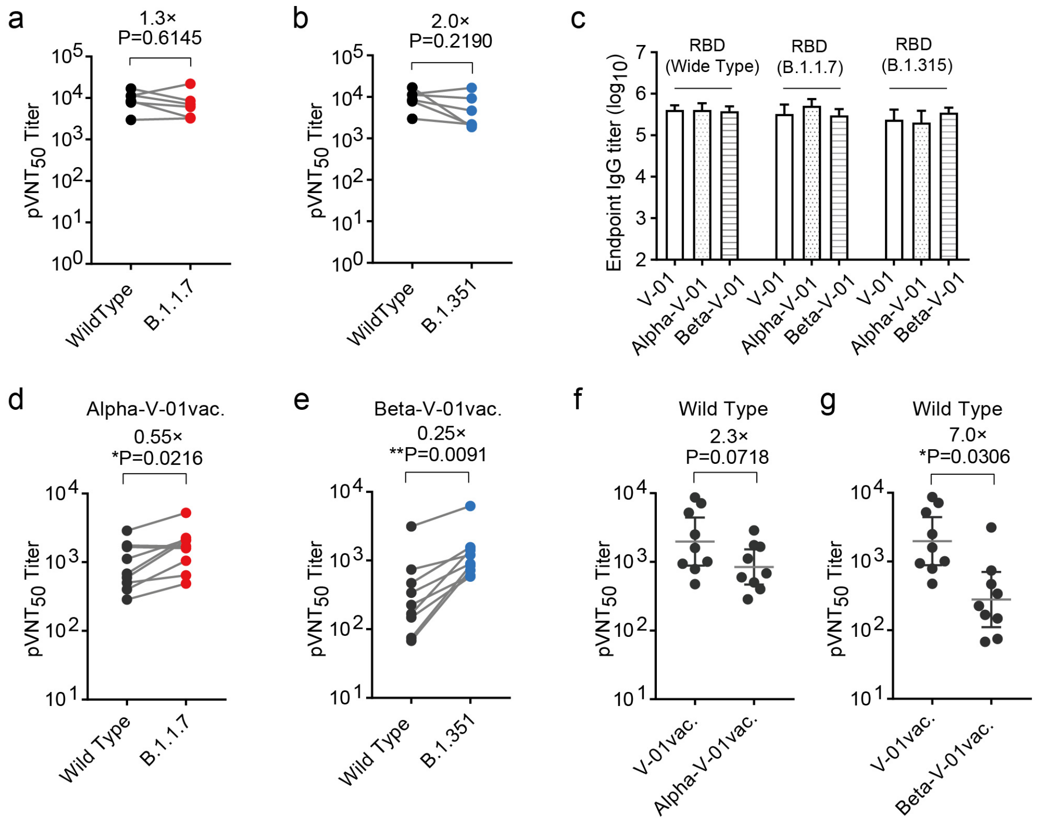

Figure S1. Analysis for the RBD-specific IgG and neutralization of variant SARS-CoV-2 by serum samples from the V-01 vaccinated mice.

a, b. C57BL/6 mice (n=6/groups) were immunized intramuscularly with 10 μg of I-P-R-F (mouse IFNα-Pan-RBD-Fc) and boosted with the same dose 14 days after the initial immunization. Serum samples were collected on day 28 after initial vaccination and used to perform the pseudovirus neutralization assays. The $pVNT_{50}$ of serum samples from mouse I-P-R-F vaccinated mice against the B.1.1.7 (a) or B.1.351 (b) variants was compared to the wild-type pseudovirus. **c.** C57BL/6 mice (n=9/group) were immunized intramuscularly with 2 μg of V-01, Alpha-V-01, and Beta-V-01 respectively and boosted with the same dose at a 14-day interval. Serum samples were collected on day 28 after initial vaccination for antibody assays. Serum IgG levels were measured by ELISA using prototype RBD, B.1.1.7 RBD, or B.1.351 RBD protein, respectively. **d.** The $pVNT_{50}$ of serum samples from the Alpha-V-01 vaccinated mice against the wild-type and the B.1.1.7 variant pseudovirus was shown and compared. **e.** The $pVNT_{50}$ of serum samples obtained from Beta-V-01 vaccinated mice against the wild-type and the B.1.315 variant pseudovirus was determined and compared. **f.** The fold-change of $pVNT_{50}$ of either V-01 or Alpha-V-01 vaccinated mice against SARS-

CoV-2 wild-type strain was analyzed. g. The fold-change of $pVNT_{50}$ of either V-01 or Beta-V-01 vaccinated mice against SARS-CoV-2 wild-type strain was determined. Factor changes in the geometric mean titer with 95% confidence intervals in the $pVNT_{50}$ titers are shown above the P values. P-values in a, b and d, e were determined using a paired two-tailed *t*-test. P-values in f and g were analyzed using an unpaired *t*-test. ns (not significant), *P<0.05, **P<0.01, ***P<0.001, ****P<0.0001.

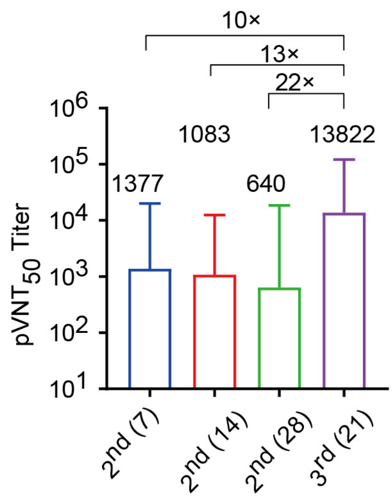

Figure. S2. Pseudovirus neutralization assays for the nAb induced by V-01 booster in rhesus macaques.

Macaques were intramuscularly immunized with V-01, as shown in figure 1c. The serum samples were collected for pseudovirus neutralization assays. The $pVNT_{50}$ against SARS-CoV-2 wild-type strain by rhesus macaque serum samples collected on day 21 after the third dose and days 7, 14, and 28 after the second dose were analyzed and compared.
